# Controlled Substance Use Among Psychiatric Patients in a Rural North Carolina Emergency Department

**DOI:** 10.5811/westjem.2018.11.40234

**Published:** 2018-12-05

**Authors:** Elizabeth Gignac, Godwin Y. Dogbey, Gregory Capece, Benjamin McMichael, Julie Aldrich, Grace D. Brannan

**Affiliations:** * Campbell University School of Osteopathic Medicine, Southeastern Health, Emergency Services, Department of Medicine, Lumberton, North Carolina; † Campbell University School of Osteopathic Medicine, Department of Clinical Research and Medical Education, Lillington, North Carolina; ‡ Campbell University School of Osteopathic Medicine, Southeastern Health, Department of Graduate Medical Education, Lumberton, North Carolina; § Campbell University School of Osteopathic Medicine, Lillington, North Carolina

## Abstract

**Introduction:**

Emergency department (ED) visits for mental health and substance use disorders have been on the rise, with substance use disorders frequently coexisting with mental health disorders. This study evaluated substances commonly used/abused by patients presenting to the ED of a rural, regional medical center with subsequent admission for mental health treatment in Robeson County, North Carolina.

**Methods:**

This retrospective, single-center study was approved by the Southeastern Health Institutional Review Board. We reviewed medical records of psychiatric patients presenting to the ED with ultimate admission to the inpatient psychiatric unit between January 1, 2016, and June 30, 2016. Frequencies of controlled substances testing positive on urine drug and alcohol screenings in admitted patients were obtained and analyzed. We also made ethnic and gender comparisons.

**Results:**

A total of 477 patients met inclusion criteria. The percentage of patients testing positive were as follows: tetrahydrocannabinol (THC) (40%); cocaine (28.7%); alcohol (15.1%); benzodiazepines (13%); opiates (9.6%); amphetamines (2.9%); barbiturates (2.3%); and methadone (0.8%). A relatively higher proportion of patients tested positive for THC than any other substance (*p*≤.0002). We found statistically significant differences for gender (*p*=.0004) and ethnicity (*p*<.0001) compositions regarding substance use/abuse.

**Conclusion:**

The majority of admitted psychiatric patients in this study tested positive for at least one controlled substance. The two substances that most often returned positive on the urine drug screen test in our sample were THC (marijuana) and cocaine. These findings may provide insight into concomitant substance abuse and psychiatric disorders, which could instigate public policy development of preventative health initiatives that explore the relationship between controlled substance use/abuse and mental health disorders in rural counties like Robeson County.

## INTRODUCTION

As the gatekeeper of the healthcare system, the emergency department (ED) serves as the safety net for most Americans, especially the uninsured, low socioeconomic status, and medically underserved populations. The ED is a primary entry point to the healthcare system for many patients who are unable to access care in outpatient centers.^1^ Patients with mental health and substance addiction issues are a particularly vulnerable population that has greater dependence on the ED for its primary healthcare needs. Prior research has shown that ED visits for mental health and substance use disorders are increasing,^1^ and that substance use disorders frequently coexist with mental health disorders.^2^ Mental health patients with substance use disorders use EDs at a higher rate than those without. Indeed, the combination of mental health issues and substance abuse contributes to the complexity of care and management of such patients.^2^ In light of this, emergency physicians and psychiatrists may need to acquire more knowledge about the issue to understand better the nuances involved in the care of this unique population.

Literature on psychiatric patients with substance use/abuse issues in underserved, rural areas such as Robeson County in North Carolina is sparse. Robeson County has some of the worst health outcomes out of all counties in the state, and its life expectancy is the lowest.^3^ It ranked 100 out of 100 counties in “Health Factors,” and 95 out of 100 counties in “Health Outcomes” in 2015.^4^ Furthermore, the Robeson County Department of Public Health designated substance misuse/abuse as one of its two top priority areas of focus during the same year.^4^ Substance use/abuse is a major contributor to morbidity and mortality in Robeson County as well as throughout the state in general. Coupled with the national trend of disproportionately increasing rates of mental health visits to EDs,^5^ we sought to better understand the relationship between mental health patients and their use/abuse of controlled substances. In this study, we explored the rate of controlled substance use/abuse among psychiatric patients who presented to the ED in a rural North Carolina regional medical center. We hypothesized that controlled substance use, as proxied by a positive urine drug screen (UDS) test, was highly prevalent (≥50%) and that ethnicity as well as gender differentials existed in the types and pattern of use of these substances among the psychiatric patients presenting to the ED.

## METHODS

### Study Population

We conducted this study at the Southeastern Regional Medical Center (SRMC) (ED), the flagship hospital for Southeastern Health in Lumberton, North Carolina. Lumberton is the county seat for Robeson County, and SRMC serves as the region’s sole comprehensive hospital. The ED is one of the busiest in the state with over 65,000 annual visits. The hospital maintains an acute inpatient psychiatric unit with 26 beds, and psychiatric professionals provide consultation for ED patients. The populations served by Southeastern Health reflect challenging characteristics that are common to many other rural communities. The hospital’s catchment area is estimated to be 950 square miles with a population of approximately 133,000.^6^ Patient demographics include a racially diverse, minority-majority population with a large Native American subpopulation. The median household income is $30,608. With a per capita income of $15,559, 30.6% of its economically disadvantaged residents live in poverty.^6^

**Population Health Research Capsule**

What do we already know about this issue?

As mental health and substance use disorders frequently coexist, emergency department (ED) visits for these associated disorders are on the rise.

What was the research question?

How high is the rate of controlled substance use among psychiatric patients in a rural ED?

What was the major finding of the study?

Over 60% of the patients admitted to inpatient psychiatry tested positive for at least one controlled substance.

How does this improve population health?

Understanding concomitant substance use and psychiatric disorders could spur early interventions to improve care for this vulnerable population.

### Design, Exclusion, and Inclusion Criteria

This study was approved by the Southeastern Health Institutional Review Board prior to the initiation of data collection. We performed a retrospective review of medical records for patients who presented to the ED at Southeastern Health and were ultimately admitted to the inpatient psychiatric unit. We included patient encounters between January 1, 2016, and June 30, 2016. We reviewed a total of 613 encounters of which 477 met the inclusion criteria. Inclusion criteria consisted of patients 18 years or older who presented to the SRMC ED and were subsequently admitted to the inpatient psychiatric unit. In addition, we included patients if they were admitted to the psychiatry department directly from the ED or admitted to a medical floor and subsequently transferred to the inpatient psychiatric unit after medical stabilization.

Inclusion criteria also required that the patient had undergone the hospital’s standard medical clearance labs: compete blood count, basic or comprehensive metabolic panel, alcohol level (EtOH), thyroid stimulating hormone level, UDS and pregnancy test (for females age 18–50). Exclusion criteria were as follows: pregnant patients, patients who had missing/incomplete data, and patients who were admitted to the psychiatric department from an outside facility. We also excluded patients who underwent psychiatric evaluation in the ED and were not admitted to the inpatient psychiatric department. The ED protocol regarding patients with primary substance abuse disorders is to refer them to a local substance abuse treatment center. They are not admitted to the psychiatry service of the hospital and thus were not included in this study.

### Data Collection, Protection of Human Subjects, and Variables of Interest

All psychiatric patients who present to the ED at SRMC undergo a standard medical screening process, which includes a UDS. Data were collected, de-identified, and entered into a Microsoft Excel spreadsheet. We stored the data on a secure flash drive and analyzed them on a password-protected computer. The following data points were collected on each patient from the medical records: age, sex, race (self-reported on registration), and the presence or absence of tetrahydrocannabinol (THC), opioids, phencyclidine, methaqualone, methadone, cocaine, benzodiazepine, barbiturates, amphetamine, and alcohol. While serum alcohol levels were measured in the ED, on data collection we recorded alcohol level as a dichotomous qualitative variable (+/−) instead of a quantitative variable for ease of data collection and analysis.

Presence of all other substances was determined by the hospital’s standard UDS. The hospital laboratory uses the Beckman Coulter® AU 5822 Clinical Chemistry System (Beckman Coulter, Inc., Atlanta, Georgia) for all UDS. The UDS instrument used at this institution does not have the capability to detect multiple opioids – both synthetic and semisynthetic. In fact, synthetic opioids such as tramadol and fentanyl are not detected by this screen. Neither are fentanyl analogs (e.g., carfentanyl) detected. The semisynthetic opioids oxycodone and buprenorphine are not detected by the screen. However, some other semisynthetic opioids, such as hydrocodone, hydromorphone, and oxymorphone, are detected.

### Statistical Analysis

We generated descriptive statistics such as frequencies/percentages for the categorical variables of interest. Means, standard deviations, and ranges were computed for the continuous variables. We performed a chi-squared test of goodness-of-fit (non-parametric test of equality of proportions across categories) in a follow-up analysis involving the most common drugs used by gender and ethnicity. Unless otherwise stated, all inferential tests were statistically significant whenever *p≤.*05. For the analyses, we used the statistical package for the social sciences (SPSS) version 24 (IBM, Chicago, Illinois) together with MedCalc Statistical Software version 16.4.3 (MedCalc Software bvba, Ostend, Belgium; https://www.medcalc.org; 2016).

## RESULTS

### Demographics

A total of 477 patients met inclusion criteria. The mean age was 37 years (±13.9 standard deviations) with values ranging from 18–97 years. There was a significant gender difference in the entire sample with more males than females (57% vs. 43%) (*p*=.004) testing positive on UDS for controlled substances. For race/ethnicity, patients who self-reported as Native American and Caucasian each made up 34.2% of the population, and 28.7% self-identified as African American, 0.2% as Hawaiian, and 2.7% as other (Table 1). This ethnicity distribution of the sample population is consistent with the ethnic/racial composition of the community. According to 2016 United Sates (U.S.) Census Bureau data, the estimated population of Robeson County was 31.3% Caucasian, 41% Native American, 24.2% African American, and 0.2% Hawaiian.^3^ Controlling for the Hawaiian and “other” categories of ethnicity/race, we found that in terms of controlled substance use reflected through testing positive on a UDS, there was no statistically significant difference in the percentages of the distribution of the major ethnicities, namely Native American (34.2%), African American (28.7%), and Caucasian (34.2%), represented in the sample (*p*=.232).

### Substance Use

The number of substances present in a psychiatric inpatient ranged from none to six, and the mean (average) number of substances that tested positive on UDS was 1.13 (±1.06 standard deviations). Furthermore, 166 (34.8%) of the 477 patients did not have a positive UDS. Conversely, 311 (65.2%) patients had at least one substance recorded on their UDS. Regarding specific substances used, THC was the most common substance for which 191 (40%) patients tested positive. Cocaine was the second most common substance, with 137 (28.7%) patients testing positive. The third and fourth were alcohol and benzodiazepines, with 72 (15.1%) and 62 (13.0%) patients, respectively, testing positive (Table 2). A positive test result for opioids was recorded on 46 (9.6%) patients, while amphetamines and barbiturates were recorded on another 14 (2.9%) and 11 (2.3%), respectively. Methadone tested positive in four (0.8%) patients’ drug screens. These results are reported in Table 2 with THC as the reference. All other substances for which patients tested positive were statistically significant among relatively smaller proportions of patients compared with THC. For example, the proportional positive test result of 40% for THC among the patients compared with that of opioids (9.6%) was significantly higher (*p*<.0001), with a 95% confidence interval of the difference between the proportions being (25.17 to 35.43).

Based on a subgroup analysis, patients testing positive for THC were more likely to be Native American (*p<.*0001) males (*p=.*001) than the other ethnic groups and gender, respectively. African-American and Caucasian males were equally as likely to test positive for THC. Similarly, patients testing positive for cocaine, the second common substance for which most patients often tested positive, were more likely to be Native American (*p<.*0001) male (*p=.*002). This trend of the results was again true for alcohol—that is, a Native American (*p<*.0001), except in this case, regardless of gender, was more likely to test positive than people of other ethnicities. Lastly, for the fourth most commonly used substance, benzodiazepine, those patients testing positive were more likely to be Caucasian (*p<*.0001) females (*p*=.042) than any of the other ethnicities in the study.

## DISCUSSION

The purpose of this study was to determine if controlled substance use (proxied by positive test result on UDS) was high among psychiatric patients who presented to the ED of a rural medical center. In addition, we aimed to examine which controlled substances were most commonly or often used (again, as gauged by a positive test result on a UDS) by this patient population. Furthermore, we sought to investigate whether ethnicity or gender differentials existed for the types and pattern of controlled substances for which positive tests resulted. Consistent with our primary hypothesis, we found that the majority of psychiatric patients, 65.2%, had at least one controlled substance in their system through testing positive on a UDS. This reflects a major public health challenge. Successful treatment of these patients likely requires attention to controlled substance use and abuse, in addition to their primary psychiatric conditions.

As indicated in the results, gender and ethnicity were significant factors relating to substance use by psychiatric patients in the study. The findings involving ethnicity are significant from a clinician’s standpoint because while Native Americans were proportionally represented as African-Americans and Caucasians in the sampled population, they tended to test positive for three of the four most common drugs observed at much higher rates than the other two groups. Such information is clinically useful as it could provide a means that alert the physician to look for warning signs during physician-patient interactions.

Findings from this study show that THC was the most common controlled substance for which patients in the study sample tested positive at a relatively higher proportion than others. The rate of THC use in the sampled population was much higher than rates reported in the general U.S. population. While it has been reported that 9.52% of U.S. adults had used THC in the prior year,^7^ 40% of patients in this study population tested positive. Some states have enacted legislation allowing THC for recreational use; however, it remains illegal in North Carolina for both medical and recreational uses. Because it is illegal, the relatively high rates of THC use may place patients at risk of legal and myriad other problems such as employment and social benefit barriers. Moreover, the long-term mental health effects of THC remain unclear in the extant literature. These highlight the importance of a holistic approach to mental health treatment, including substance abuse education, treatment, and management.

Cocaine was the second most frequently used substance reported with 28.7% of patients testing positive. This rate is dramatically higher than the rate of cocaine use/abuse by the general U.S. population. Studies have shown that 0.6% of U.S. adults reported using cocaine within the prior 30 days.^8^ One potential reason for the high levels of cocaine use in the study population is its geographic location. Robeson County is located halfway between New York and Miami on Interstate 95. Rural North Carolina locations often serve as temporary cocaine storage sites for criminal groups as they move the product from one region to another.^9^ Robeson County’s rural nature and proximity to a major highway makes it an ideal site for cocaine storage and distribution.

The U.S. Department of Justice, has estimated that 75–80% of the cocaine in North Carolina is distributed as crack cocaine.^9^ Due to the economic disparity of our population, this is presumed to be the primary form used by our patients. The estimated price of powdered cocaine is $100 per gram, while crack cocaine is sold for approximately $10–25 per rock.^9^ As cocaine use induces changes in neurotransmitters such as dopamine and glutamate, its use complicates mental health treatment. Studies show that chronic cocaine use leads to impairment in cognition and stress management, and can lead to increases in anxiety, irritability, paranoia and psychosis.^8^ These highlight the necessity of a multi-faceted approach to mental health care in the study population.

Alcohol was present in the serum of 15.1% of patients studied. However, no data were available for overall alcohol use/abuse rates in the county. Alcohol acts as a central nervous system depressant and is commonly used by patients with psychiatric conditions. Alcohol intoxication and withdrawal affect numerous neurotransmitters including gamma-aminobutyric acid, dopamine, N-methyl-d-aspartate, adrenocorticotropic hormone, and endorphins.^10^ Patients who use alcohol are at risk for myriad psychiatric problems including depression, anxiety, hallucinations, impaired judgment, and impaired cognition.^10^ In addition, the concurrent use of alcohol with prescription medications can lead to numerous adverse effects. Screening for alcohol use and abuse is essential in the treatment of patients with psychiatric complaints, as alcohol treatment and mental health treatment are codependent entities. The success of one depends on the other.

Benzodiazepines were present in 13% of patients studied. One of the limitations of this study is that we were unable to determine when benzodiazepines were ingested and for what purpose. Benzodiazepines serve as a therapy for certain psychiatric conditions such as anxiety and panic disorders. They are also frequently administered to acutely agitated patients in the ED. Administration of benzodiazepines in the ED occasionally occurs prior to the collection of the patient’s urine specimen for the UDS. When this occurs, the patient’s urine will test positive for ED-administered benzodiazepines. Conversely, abuse of benzodiazepines is not uncommon. Benzodiazepines may be abused to potentiate the effects of other drugs and are sometimes misused to mitigate withdrawal symptoms from other substances.^11^ As stated, we were unable to determine the exact role that benzodiazepine use plays in the patient population, but screening for benzodiazepine use and abuse should be included in all comprehensive psychiatric treatment regimens.

Most surprisingly, opioids were found to be the fifth most common controlled substance found in the patient population. Opioids were present in 9.6% of patients, and methadone was present in an additional 0.8% of patients. This finding is lower than expected, given the nation’s current opioid epidemic. In 2016, the same year in which the data for this study was collected, the Centers for Disease Control and Prevention reported that 42,249 Americans died from opioid overdoses.^12^ Similarly, North Carolina reported 1,956 opioid overdose deaths in 2016.^12^ As evidenced by the number of opioid-related deaths, the opioid crisis exists throughout the entire state of North Carolina.

In Robeson County, 1.476 opioid prescriptions were written per resident in 2016, and the statewide average was 1.06—almost 47% above the state average.^13^ Additionally, there were 113.3 opioid pills per resident prescribed in the county in 2016. This is higher than the statewide average of 78.3 pills per person.^13^ Although opioid abuse remains a national and local health crisis, only a small percentage of the study patients tested positive for opioids in their UDS. Furthermore, it is uncertain whether the opioids detected in these patients represented therapeutic use, misuse or illegal use. It is also worth noting that the majority of ED patients with primary substance abuse disorders are referred to substance abuse treatment centers and are not admitted to the psychiatric service unless they have a primary psychiatric condition.

In North Carolina and throughout the U.S., we must caution against over-interpretation of the relatively low prevalence rate of opioid use in our study population. Indeed, the drug-screening kit used in this study does not detect the presence of the semi-synthetic opioid oxycodone or synthetic opioids tramadol, buprenorphine, fentanyl, and fentanyl analogs (e.g., carfentanyl). A growing body of evidence suggests that fentanyl and synthetic opioids account for a substantial proportion of opioid use and abuse. In 2016, 47% of all opioid-related deaths in the U.S. were attributed to use/abuse of synthetic opioids other than methadone.^15^

Numerous fentanyl products are available by prescription, and fentanyl and other novel synthetic opioids are sold illegally on the streets. Often, synthetic opioids may be erroneously marketed on the streets to unsuspecting users as heroin or other narcotics.^16^ Synthetic opioids may be found in powdered or pill form, and may be smoked, injected, snorted, or ingested by the user.^17^ These synthetic opioids are not detected by the commonly available, commercial UDS kits such as the one used used in this study. Hence, the prevalence of their use in the study population is not empirically well known. Consequently, we would suspect that the rate of opioid use could be significantly higher than the 9.6% observed among the population studied.

## LIMITATIONS

No causation or correlation can be adduced from this study, but it could provide useful insights that serve as a foundation for future studies for a rural, healthcare-needy, underserved, and vulnerable population. There are several limitations to this study, the first of which relates to interpretation of UDS in general. Each drug tested is detectable in the urine for different periods of time. Given this, it is very difficult to accurately obtain and compare the true prevalence of one drug to another.^14^ Moreover, certain commonly abused opioids, including fentanyl and tramadol, are not reliably detected with the test machine used at this facility. Synthetic amphetamines and benzodiazepines are also not detected. Therefore, the use of these drugs may be more prevalent in the population than reported here.

This study was conducted at a single medical center in a rural area where the demographics may not be fully representative of all counties in North Carolina. Also, because patients with acute psychosis may require some of the medications measured for agitation they may not, in a real sense, be abusing those substances. When administered prior to urine collection, this could lead to a positive drug screen. As mentioned previously, there were also several patients who must have been prescribed benzodiazepines and/or opioids pain medications on an outpatient basis. These patients who were more likely to test positive on presentation to the ED might have been included in this study despite not necessarily qualifying as substance-abusing subjects. Indeed, we were unable to obtain accurate data on those cases from the medical records to determine the number of patients who might have been legally prescribed medications that would have led to a positive controlled-substance screen result. The electronic medical records reviewed indicated that many patient encounters had missing or incomplete home medication lists. Furthermore, for patients who were administered benzodiazepines screening in the ED, the timing of the urine collection was not consistently documented. Patients who received benzodiazepines prior to collection of their urine would likely have a positive UDS result for benzodiazepines.

The short duration of the time span or the relatively short period reviewed for the study may have limited the observance of greater prevalence of drug use/abuse in the subpopulation of patients studied. Hence, prevalence may be underestimated. Despite these limitations, our results provide baseline information that could trigger conversations among healthcare stakeholders to devise ways to intervene to improve the health of this unique population nationwide.

## CONCLUSION

This study highlights that mental health and substance use disorders frequently coexist. In the rural area studied, over 60% of patients admitted to inpatient psychiatry tested positive for one or more controlled substances. While our findings may not necessarily reflect accurate drug usage rates due to the increasing use of synthetic opioids, which are not easily detectable with many UDS kits, these results may provide insight into concomitant substance abuse and psychiatric disorders in rural areas. Ideally, this study will spur local, state, and federal agencies to look more closely at the relationship between substance use and mental health disorders and guide them in developing preventative health initiatives and allocating requisite resources to help mitigate substance abuse, especially in these underserved areas of need. Ultimately, our study suggests the need for multiregional, longitudinal studies to examine the substance abuse rates as well as patterns in psychiatric populations in various regions and differing socioeconomic strata. Most importantly, future studies should be able to differentiate legal uses from cases of actual substance abuse.

## Figures and Tables

**Table 1:** Patient demographics (N=477).

Characteristics	N	%	P
Gender/sex			.004
Female	207	43.6	
Male	270	56.6	
Race/ethnicity			< .0001
American Indian	163	34.2	
African American	137	28.7	
Caucasian	163	34.2	
Hawaiian	1	0.2	
Other	13	2.7	

**Table 2:** Substance use profile — distribution by the type of substance abused by patient (N = 477).

Substance	N*	%*	P**	95% CI (LL%, UL%)
THC (marijuana)	191	40.0	reference	reference
Opioids	46	9.6	< .0001	(25.17, 35.43)
Phencyclidine	0	0	n/a	n/a
Methaqualone	0	0	n/a	n/a
Methadone	4	0.8	< .0001	(34.71, 43.69)
Cocaine	137	28.7	.0002	(5.28, 17.21)
Benzodiazepine	62	13.0	< .0001	(21.6, 32.2)
Barbiturate	11	2.3	< .0001	(33.05, 42.27)
Amphetamine	14	2.9	< .0001	(32.39, 41.71)
Alcohol	72	15.1	< .0001	(19.36, 30.24)

*CI*, confidence interval; *THC*, tetrahydrocannabinol; *n/a*, not applicable; *LL*, lower limit; *UL*, upper limit

* These Ns total more than 477 because of multiple choices of substance types. The percentages were based on the 477 original total.

** P values were based on the difference of proportion of substances used with THC as reference.

na = not applicable.
